# Hypersensitivity Reaction and Tolerance Induction to Ethambutol

**DOI:** 10.1155/2013/208797

**Published:** 2013-07-28

**Authors:** Josefina Rodrigues Cernadas, Natacha Santos, Claudia Pinto, Patricia Caetano Mota, Mariana Castells

**Affiliations:** ^1^Immunoallergy Department, Centro Hospitalar Sao Joao, E.P.E., 4200-319 Porto, Portugal; ^2^Pulmonology Department, Centro Hospitalar Tras-os-Montes e Alto Douro, E.P.E., 5000-508 Vila Real, Portugal; ^3^Pulmonology Department, Centro Hospitalar Sao Joao, E.P.E., 4200-319 Porto, Portugal; ^4^Department of Medicine, Division of Rheumatology, Immunology, and Allergy, Brigham and Women's Hospital, Boston, USA

## Abstract

Tuberculosis remains the leading cause of death worldwide from any infectious agent and the alarming increase in the annual incidence of new cases has been described as a global emergency. *Mycobacterium* infection requires simultaneous administration of multiple drugs. Although the majority of treatment courses progress with minor side effects, adverse reactions to antituberculosis drugs occur in about 5% of treated patients and can be responsible for cessation or switching the therapy. Both nonimmediate (mostly maculopapular rash) and immediate reactions (urticarial reactions) have been described with these drugs. The main problem is the occurrence of reactions while the patient is on treatment with multiple drugs. The diagnosis of the culprit drug is mostly based on stopping all medication, followed by the reintroduction of each drug with a time interval of four to five days. An alternative drug should be the first approach if it is equally effective. Most of the times, none of the alternative drugs are as effective as the culprit. If this is the case, a desensitization procedure should be performed. The authors describe a case of a woman with *Mycobacterium avium* complex (MAC) infection, to whom treatment with ethambutol was crucial to recovery, and present a modified desensitization protocol to this drug.

## 1. Case Report

We present the case of a 66-year-old woman with a history of hypertension, dyslipidemia, chronic gastritis, depression, and non-cystic fibrosis bronchiectasis, followed in our hospital's Pulmonology Department. Her medication was losartan 100 mg with hydrochlorothiazide 25 mg id, omeprazole 20 mg id, ticlopidine 250 mg id, sertraline 50 mg id, and inhaled fluticasone 500 ug with salmeterol 50 ug bid. In 2006, she was diagnosed with atypical mycobacteriosis with* Mycobacterium kansasii*, having completed 18 months of rifampicin, ethambutol, and isoniazid without adverse reactions. In May 2008, *Mycobacterium avium* complex (MAC) was identified in two sputum cultures, for which she started streptomycin (2 months), rifampicin, ethambutol, and clarithromycin. After 14 months of treatment, she presented facial erythema and angioedema of the neck and upper limbs followed by pruritic scaling maculopapular exanthema in the neck, trunk, and upper and lower limbs, associated with mild dyspnoea with no hemodynamic changes. She recurred to the hospital and was treated with intravenous antihistamines and corticosteroid. All medication was stopped, with complete resolution in 1 week. Because MAC persisted in cultures, treatment was started with rifampicin and clarithromycin for 1 month, followed by 400 mg of ethambutol. Despite premedication with oral hydroxyzine, she had a similar reaction 3 hours after ethambutol intake. Ethambutol was stopped and she was treated with oral hydroxyzine for two days, with complete resolution in one week.

Despite treatment with rifampicin and clarithromycin, MAC persisted in sputum cultures until November 2010. At this point, the patient was referred to the Drug Allergy Unit. 

Skin tests were performed according to EAACI guidelines [1], using solutions of 1 mg/mL and 10 mg/mL [2] prepared from a 400 mg ethambutol tablet. Immediate reading was negative, but a flare reaction with the 1 mg/mL concentration was present at 6 hours, which resolved at 24 and 72 hours readings. Although skin testing was doubtful, hypersensitivity to ethambutol was diagnosed taking into account the clinical presentation and reproducibility of the reaction upon reexposure.

A two-day tolerance induction protocol was attempted in an outpatient regime, adapted from previous literature reports [3], with a twofold increase every 45 minutes. The first day was completed with 31.6 mg of cumulative dose, with no adverse events. The second day initial dose was 16 mg, and at a cumulative dose of 130 mg, the patient had a similar reaction to the previously described one. The procedure was modified according to current recommendations [4] to achieve 1200 mg in four days, without success.

As ethambutol was the optimal choice for treatment of MAC, a second protocol was designed based on the twelfth step protocol by Castells et al. [5] (Table 1), with slower increments and premedication with 25 mg of hydroxyzine and 20 mg of oral prednisone. The patient reached 400 mg in the first day and continued on 400 mg during 8 days. Increases to 800 mg and to 1200 mg were done after one week in each dose without adverse reactions. Prednisone and hydroxyzine were discontinued and she was maintained on ethambutol 1200 mg daily for 6 months with good tolerance. In April 2011, the first sputum culture turned negative, but she was unable to complete a full-year treatment due to a confirmed diagnosis of ethambutol-induced optic neuropathy in June 2011. In July 2011 she was diagnosed with hepatic adenocarcinoma of unknown primary and *Aspergillus fumigatus* was isolated from sputum cultures. Ethambutol was not reintroduced and all antimycobacterial treatment was stopped. The patient is currently followed in Palliative Care, as well as by the Pulmonology Department.

## 2. Comments

Ethambutol is a bacteriostatic antimycobacterial drug that has ocular toxicity due to optic neuritis as the main side effect. Reactions as rash and drug fever have been reported in 0.5% and 0.3% of patients, respectively [6], and other hypersensitivity reactions, as ashy dermatosis-like pigmentation [7], lichenoid eruptions [8], pulmonary infiltrates [9], and toxic epidermal necrolysis [10], have been described. Ethambutol is usually used in combined therapy with other antibiotics, which makes diagnosis particularly difficult. The recommended approach in generalized cutaneous reactions is stopping all combined treatment and gradually reintroducing each of the antimycobacterial drugs, to determine the offending drug [11]. Skin and serological tests are usually unreliable, with one paper presenting a positive epicutaneous test [12] and another positive lymphocyte stimulation test [9] to ethambutol.

We present a case of a clinically confirmed hypersensitivity reaction to ethambutol in whom a previously described protocol was ineffective. This new adapted successful desensitization protocol underscores the importance of lower incremental doses with more steps and the use of antihistamines and prednisone to induce immunological tolerization. Because the mechanism of the initial reaction appears to be a hypersensitivity type IV reaction, these reactions have been shown to be responsive to a slower reintroduction of the offending agent, so that reaching the target dose may require few weeks. The mechanism of desensitization may relate to a suboptimal T cell antigen presentation. 

## Figures and Tables

**Table 1:** Desensitization protocol for ethambutol.

Solution (mg/mL)	Time (min)	Dose (mL)	Dose (mg)	Cumulative dose (mg)
0.01	0	1	0.01	0.01
	30	2	0.02	0.03
	60	4	0.04	0.07
	90	8	0.08	0.13

0.1	120	1	0.1	0.23
	150	2	0.2	0.43
	180	4	0.4	0.83
	210	8	0.8	1.63

1	240	1	1	2.63
	270	10	10	12.63

10	300	10	100	112.63
	330	30	300	412.63
